# Mental health in the pandemic

**DOI:** 10.2471/BLT.21.020921

**Published:** 2021-09-01

## Abstract

The psychological repercussions of COVID-19 have engendered multiple ad hoc initiatives and raised awareness of the need for investment in mental health services. Tatum Anderson reports.

For Dr Mythili Hazarika, India’s first national lockdown was above all a psychosocial event. “It was like a massive psychological experiment,” she says. “People found themselves suddenly without incomes and forced to stay home in often cramped and unsanitary housing. Others, including mothers of newborns, were deprived of essential health services, medicines and food.”

An associate professor of clinical psychology in the state of Assam, and for 20 years the only clinical psychologist in a public hospital in Guwahati, the state capital, Hazarika was unable to gauge the size of the problem, but reports from patients and colleagues convinced her it was substantial. So much so that she felt she had to do something.

What she did was call for the setting up of a state-wide mental-health help line. The Assam police were the first to respond. For Hazarika this came as no surprise. “In many instances the police are the first to deal with the consequences of mental illness, including domestic violence, drug overdoses and suicides and we work closely with them,” she says.

By April 2020 the police had provided six phone lines for the initiative. Subsequently, and after what Hazarika describes as multiple negotiations, Assam’s state health department got involved, agreeing to repurpose an existing health helpline service, and ‘Monon: Assam Cares’ was born.

In May 2021, some 400 volunteers from across India, trained by Hazarika and her colleagues in batches of 50 to 60 via webinar, started to listen to what the people of Assam were saying. And what they heard was shocking.

“It was like an emotional tsunami,” Hazarika says. “People were desperate. Some told stories of penury and hunger, others talked about violence and abuse, often linked to forced confinement. Some had reached breaking point and were threatening to throw themselves off the Saraighat bridge (a bridge in Guwahati that has been the location of many suicides).”

Monon’s volunteers soaked up the incoming calls but they also reached out, calling patients diagnosed with coronavirus disease 2019 (COVID-19) quarantined within Assam’s isolation centres. In the first six months, they contacted some 43 700 COVID-19 infected people, providing what Hazarika refers to as psychological first aid, a mixture of active listening (a listening technique that includes the generation of paraphrased feedback) and problem-solving therapy (providing patients with the tools needed to identify and solve problems) as well as meditation and relaxation techniques.

Volunteers also provided grief counselling to families who lost family members to the pandemic and, where necessary, referred patients to hospitals, often collaborating with the police to intervene. According to Hazarika, the work took a considerable toll on the counsellors who themselves often needed psychosocial support.

“It was like an emotional tsunami.”

Mythili Hazarika

Monon is just one example of initiatives launched worldwide, as communities struggle to cope with the mental health repercussions of a pandemic exacerbating risk factors such as unemployment and financial instability, disruption to education, social isolation, intimate partner and family violence, fear of life-threatening disease, and sudden loss of loved ones.

In Nigeria, for example, authorities in Lagos state hired counsellors to staff COVID-19 isolation units and started a toll-free mental health line in April 2020, once again inspired by a local psychologist – Dr Aisha Abdullahi – who started a free online counselling service after seeing a rise in COVID-19 related mental health case reports.

Nigeria-based nongovernmental organization Mentally Aware Nigeria Initiative (MANI) also got involved, building on work it had been doing since 2016. According to Dr Victor Ugo, MANI’s founder, MANI’s crisis and counselling centre saw a 70% increase in the number of people calling, rising from around 1000 to 1700 people during the first three months of the pandemic. As in Assam, the counsellors themselves suffered mental health consequences. “Counsellors were breaking down,” Ugo says. “We had to get help from partners in South Africa.”

While such efforts are clearly admirable, they beg questions regarding the level of mental and psychosocial support being provided by national governments during the pandemic.

Dr Fahmy Hanna, a technical officer working in the World Health Organization (WHO) Department of Mental Health and Substance Use, is somewhat encouraged by the fact that 90% (108 of 120) Member States responding to a WHO pulse survey in early 2021 self-reported that mental health and psychosocial support were included in their COVID-19 response plans.

But he is the first to point out that the same survey found that mental health provision was the most disrupted of all health services during the pandemic. “Specialist facilities and workers were often re-assigned, with the full or partial closure of services, including outpatient, emergency and counselling services in 45% of the 121 countries reporting,” he says.

In some cases, fear of virus transmission has led to facilities closing their doors. According to MANI’s Ugo, this was the case in some Nigerian psychiatric hospitals that ran out of personal protective equipment. “Hospitals stopped taking admissions and in some cases even discharged most of the patients from the wards,” he says. “Health workers were not keen to put themselves at risk of exposure to the virus, especially where they were lacking appropriate PPE.”

Many countries cut funding for mental health services as part of a broader reallocation of health spending to boost pandemic response efforts. According to the WHO pulse survey, just 20% of countries fully allocated the budget required for implementation of mental health and psychosocial support programmes in 2021.

For Hanna, even more worrying is the fact that 23% of countries were actually reducing community-based mental health services during the pandemic. “Even before the pandemic, average government mental health expenditure was less than 2% of total health expenditure and most of that (around 60%) goes to mental hospitals. At a time when we are talking about scaling up these services, countries are scaling down,” Hanna says.

Daisy Fancourt, a psychobiologist and epidemiologist at University College London, expresses similar concerns about the overall investment picture. “Pre-pandemic an estimated third to a half of the global population experienced mental health problems, yet just 5% receive evidence-based treatments. The economic impact of the pandemic is causing some governments to reconsider priorities so that even that low level of service provision is in doubt.”

“The mental health impacts of the post-COVID condition, bereavements, job losses and poverty are going to be felt for several years.”

Daisy Fancourt

Fancourt founded the COVID-Minds Network, a project bringing together 150 longitudinal studies on mental health in more than 70 countries, including a real-time UK study of 72 000 people that has been running since the pandemic began. The project has generated a number of intriguing findings, including the fact that suicide levels have not risen in most countries and that reactions to stressors such as quarantining have varied from country to country, with some countries reporting a net improvement in mental well-being, notably where there was a perception that decisive action had been taken and that everyone was being confined.

Nevertheless, Fancourt believes it is going to be vital to boost and sustain mental health service provision to meet increased demand in the coming year. “The mental health impacts of the post-COVID condition, bereavements, job losses and poverty are going to be felt for several years,” she says.

Fancourt hopes that increased awareness of mental health challenges developed during the pandemic will help advance the mental health agenda going forward. Hanna points to early signs that this may already be happening. “The inclusion of mental health as an agenda item for the first time at the World Health Assembly [which was held in May 2021] is a significant step,” he says.

Hanna also points to the extension of the Mental Health Action Plan (2021–2030) which was endorsed by the Health Assembly and includes a new target for mental health and psychosocial support in emergency preparedness and response. WHO is supporting the development of emergency response capacity, notably collaborating with standby partners in the development of the first-ever programme for rapid deployment of experts in mental health and psychosocial support during public health and humanitarian emergencies.

Whether high-level discussion, strategizing and target-setting feeds through to greater prioritization of mental health services within national health budgets remains to be seen. MANI’s Ugo has doubts, citing historic neglect and persistent mental health stigmatization. “Politicians may pay lip service to new mental health agendas, but so far the commitment hasn’t been there,” he says.

Hazarika is similarly realistic. While pleased to see that the Assam authorities launched its own version of the Monon service in June – albeit with just 39 counsellors – she laments the lack of investment in mental health at the state and national levels. “Hopefully the pandemic will force the government to pay attention,” she says. “If it doesn’t you have to ask yourself what will.”

**Figure:**
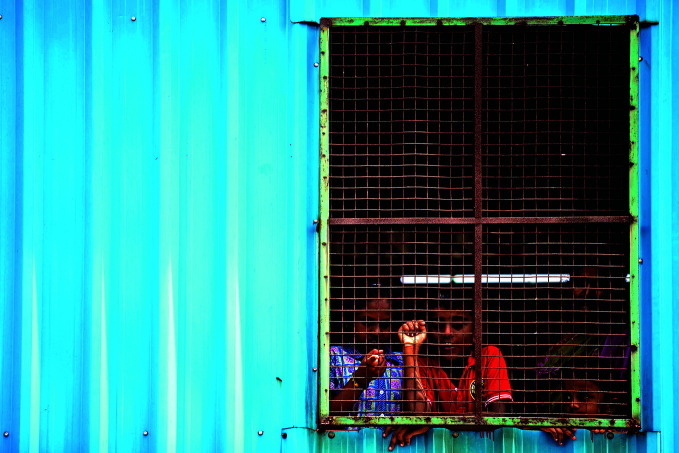
A family living under COVID-19 lockdown in Tamil Nadu, India

UNICEF/Srishti Bhardwaj

**Figure:**
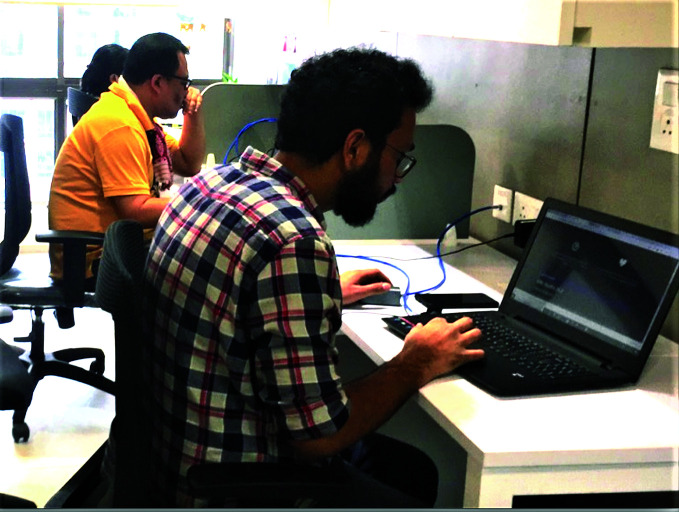
Monon volunteers calling Covid-19 patients to offer psychosocial support.

Courtesy of Mythili Hazarika

